# Supplementary material for: Galectin-3-ITGB1 Signaling Mediates Interleukin 10 Production of Hepatic Conventional Natural Killer Cells in Hepatitis B Virus Transgenic Mice and Correlates with Hepatocellular Carcinoma Progression in Patients
Source: Viruses. 2024 May 7;16(5):737. doi: 10.3390/v16050737 (PMC11125742; doi:10.3390/v16050737)
Supplement: Supplementary file 1 [file viruses-16-00737-s001.zip › viruses-2964059-supplementary.pdf]

Supplemental materials and methods

Antibodies for flow cytometry analysis

Antibodies for flow cytometry analysis included APC-Cy7-CD45, APC-CD3, PE-Cy7-CD3, APC-Cy7-CD3, BV605-NK1.1, Percp-Cy5.5-IFN-γ, FITC-CD29, Percp-Cy5.5-Rat IgG1,κ, and PE-Rat IgG2b,k (BioLegend, San Diego, CA, USA); BUV395-CD3, BV421-CD49b, APC-CD49b, BV786-CD49a, BUV395-CD49a, FITC-CD69, PE-PD-L1, FITC-CD107a, PE-CD107a, PE-IL-10, BV786-IFN-γ (XMG1.2), FITC-Rat IgG2a κ, PE-Rat IgG2a κ, PE-Rat IgG2a λ, APC-Rat IgG1κ, and BV786-Rat IgG1 κ (BD Biosciences, San Diego, CA, USA). APC-NKG2D, PerCP-eFluor 710 -TIGIT, Percp-Cy5.5-LAG-3, and PE-galectin-3 (eBioscience, San Diego, CA, USA).

mRNA sequencing

The isolation of primary mouse hepatocytes was performed as described [47]. Total RNA was extracted from the purified hepatocytes using a miRNeasy Mini Kit (QIAGEN, Duesseldorf, Germany). Illumina paired-end 150-bp sequencing was performed using a HiSeq2500 apparatus (Illumina, San Jose, CA, USA). Differentially expressed genes (DEGs) (fold change≥2) were converted to Entrez-IDs for GO analysis with R 3.2.3 software using the R Bioconductor genome-wide mouse annotations from package org.Mm.eg.db (version 3.4.0) (Author: Marc Carlson). The results were ranked according to the p values ($p<0.05$).

Quantitative Real-time PCR

Total RNA of liver tissues was extracted using TRIzol reagent (Invitrogen, Carlsbad, CA, USA). mRNA was reverse transcribed into cDNA in an 80 μL reaction volume containing the following reagents: 8 μg of total RNA, 5 μmol/L oligdT, 0.5 mmol/L dNTPs, 16 μL of 5× buffer, 10 mmol/L DTT, 160 units of RNase inhibitor, 800 units of M-MLV and distilled water (ultrapure, DNase and RNase free). Total RNA was extracted from the purified NK cells ($CD3^-$ $NK1.1^+$) using a miRNeasy Mini Kit (QIAGEN, GmBH, Germany). mRNA was reverse transcribed into cDNA in a 40 μL reaction volume containing the following reagents: 1 μg of total RNA, 5 μmol/L oligdT, 0.5 mmol/L dNTPs, 16 μL of 5× buffer, 10 mmol/L DTT, 80 units of RNase inhibitor, 400 units of M-MLV and distilled water (ultrapure, DNase and RNase free). The RT reaction was performed at 37°C for 50 minutes, followed by heating at 70°C for 15 minutes. The 20 μL volume reaction contained 10 μL 2×PCR buffer, 2 μL cDNA template, 0.4 μmol/L forward and reverse primers. Quantitative real-time PCR was performed using a Roche Light Cycler 480 II (Roche Diagnostics, Germany). PCRs were performed using a total of 45 cycles consisting of a 20 s melt at 95°C, followed by a 30 s annealing at 58°C, and 50 s extension at 72°C. The primers used were as followed. Lgals3 primer: forward (P1) 5′-GAAACCCAACGCAAACAGGA-3′; reverse (P2) 5′- TTGACCGCAACCTTGAAGTG-3′; product: 228 bp. IL-10 primer: forward (P1) 5′-GGTGAGAAGCTGAAGACCCT-3′, reverse (P2) 5′-TGTCTAGGTCCTGGAG-TCCA-3′; product: 248 bp. β-actin primer: forward (P1) 5′- TGACGTTGACATCCG-TAAAGACC -3′, reverse (P2) 5′- CTCAGGAGGAGCAATGATCTTGA -3′; product: 148 bp. Each sample was analyzed in triplicate for each target gene. The results were analyzed using the ΔΔCt method.

Cytotoxicity of cNK cells against tumor cells

Purified cNK cells ($1×10^6$ /mL) were added with recombinant mouse Gal-3 protein (1 μg/mL, R&D Systems, Minneapolis, MN, USA) and cultured in PBS at 4 °C for 30 min. Yac-1 cells ($1×10^5$ /mL, ATCC, Manassas, VA, USA) were resuspended in RPMI 1640 with 10% FBS, and then stained with a CellTrace™ Far Red Kit (Invitrogen, Carlsbad, CA, USA). Gal-3-treated hepatic or splenic cNK cells ($1×10^5$/100 μL) and stained Yac-1 cells ($1×10^4$/100 μL) at an E:T ratio of 10:1 were plated into 96-well round-bottom plates (200 μL/well) and cultured in RPMI 1640 with 10% FBS for 4 h at 5% CO2 in a 37 °C incubator.

Four hours later, the cells were harvested for validity detection. The maximum release was detected for target cells by 0.05% Triton-100 in 10% FBS RPMI 1640, and spontaneous release was detected for target cells by 10% FBS RPMI 1640.

Histological examination

Liver samples were fixed in 10% neutral-buffered formalin and embedded in paraffin. Sections (4 μm) were stained with hematoxylin and eosin using routine methods. The sections were photographed using a Pannoramic MIDI or SCAN (3D HISTECH, Budapest, Hungary). Rabbit anti-Galectin 3 antibody (ab 53082, Abcam, Cambridge, MA, USA), mouse anti-IL-10 antibody (sc-1783, Santa Cruz Biotechnology, Santa Cruz, CA) and biotinylated anti-mouse IgG (PV6000, ZSGB-bio, Beijing, China) were used for the immunohistochemistry staining according to the manufactures' instructions.

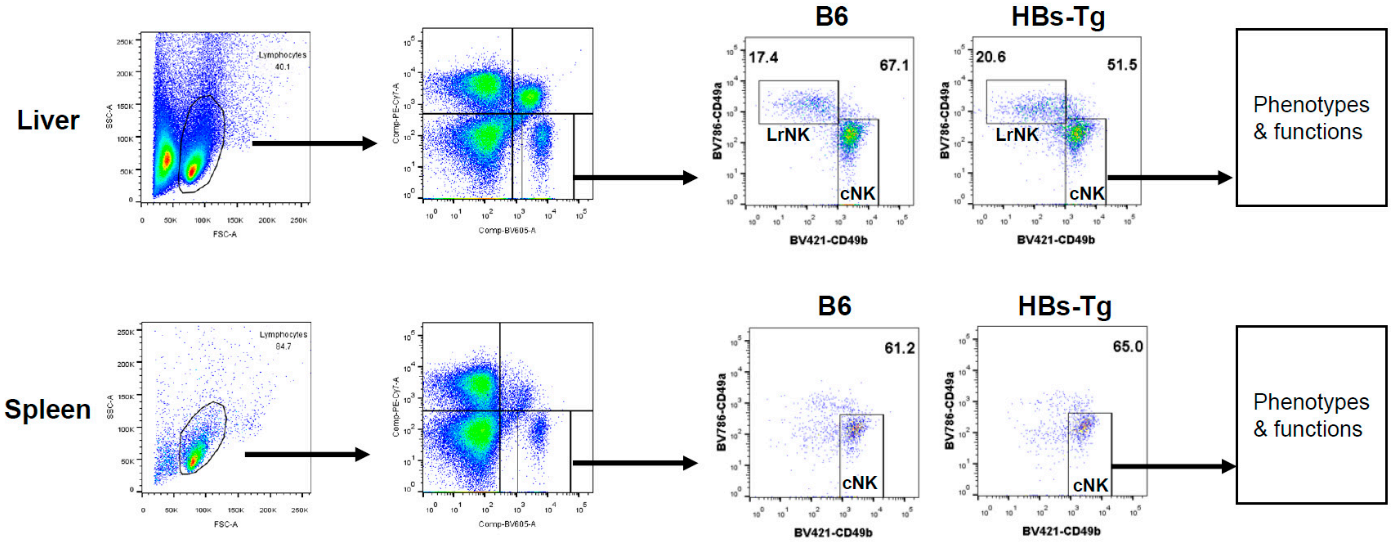

Figure S1. Gating strategy for NK cells in the liver and spleen for flow cytometry analysis. MNCs were isolated from livers and spleens of HBs-Tg mice and control WT B6 mice, and then analyzed by flow cytometry. MNCs were gated according to FSC and SSC, and total NK cells ($CD3^{-}NK1.1^{+}$) were then gated in the liver and spleen, respectively. LrNK ($CD3^{-}NK1.1^{+}CD49b^{-}CD49a^{+}$) and cNK cells ($CD3^{-}NK1.1^{+}CD49b^{+}CD49a^{-}$) were gated for the analysis of phenotypes & functions.

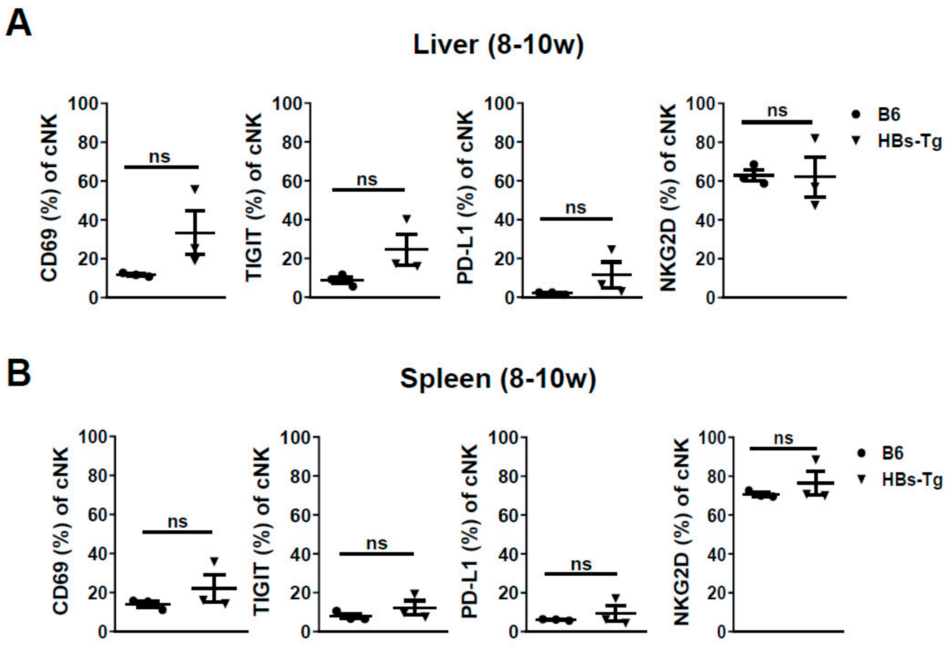

Figure S2. No significant differences were observed in the phenotypes of cNK cells in the liver and spleen of 8-10-week-old HBs-Tg mice. MNCs were isolated from livers and spleens of 8-10-week-old HBs-Tg mice and control WT B6 mice, and then analyzed by flow cytometry. cNK cells ($CD3^-$ $NK1.1^+CD49b^+CD49a^-$) cells were gated to analyze the expression of phenotypical molecules for livers and spleens. Data are shown as mean ± SEM. Student's *t* test was used. No significant statistical difference was defined as ns.

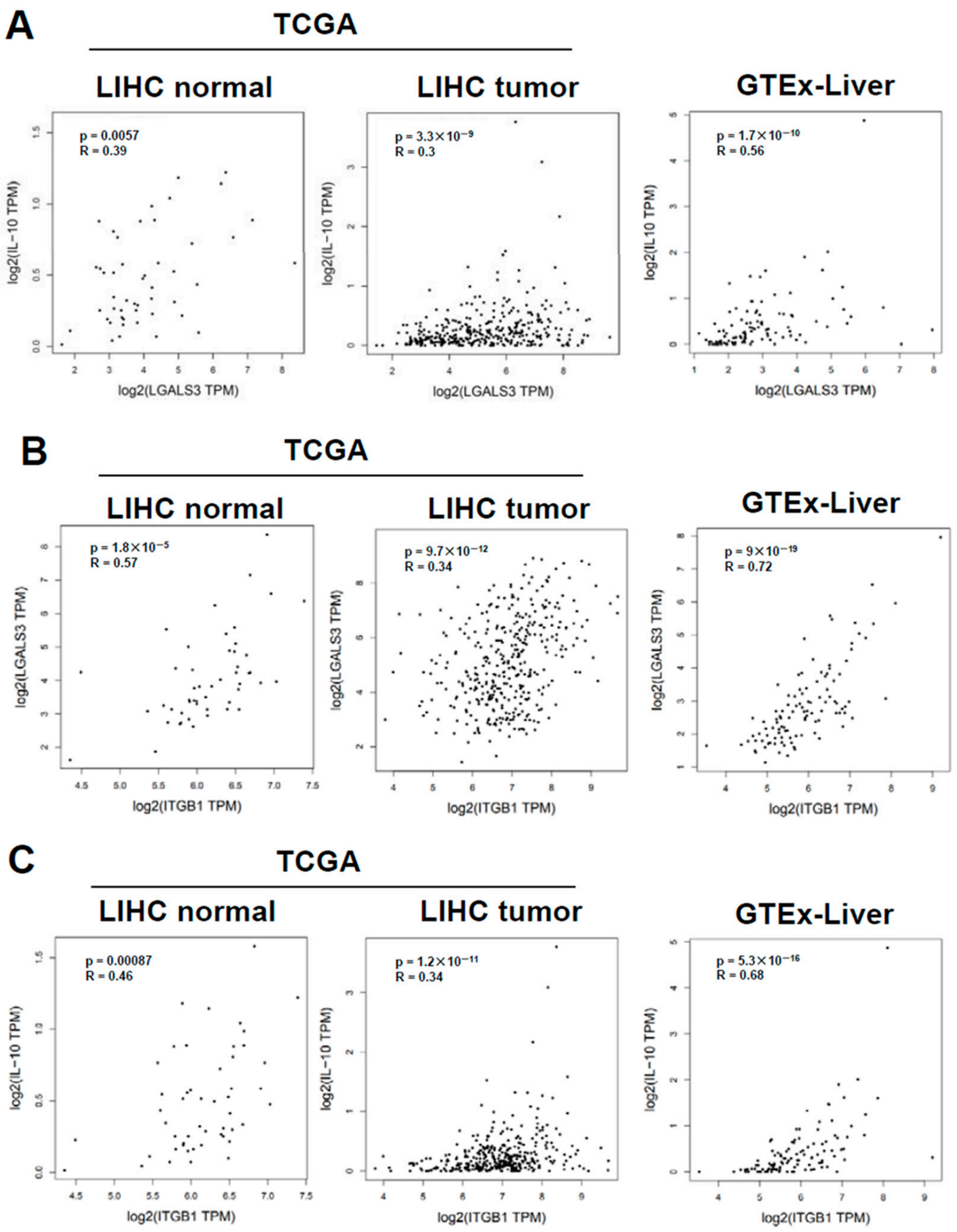

Figure S3. LGALS3 and ITGB1 expressions correlated to IL-10 expression in human liver tissue. Gene Expression Profiling Interactive Analysis (GEPIA2) was used to perform the correlation analysis for given sets of TCGA and GTEx expression data. Correlation Coefficient was analyzed using Spearman. Liver hepatocellular carcinoma (LIHC) Normal (50 samples) and Tumor (369 samples), and GTEx liver (110 samples) were included. (**A**) Correlation analysis of two genes LGALS3 and IL-10. (**B**) Correlation analysis of two genes ITGB1 and LGALS3. (**C**) Correlation analysis of two genes ITGB1 and IL-10.

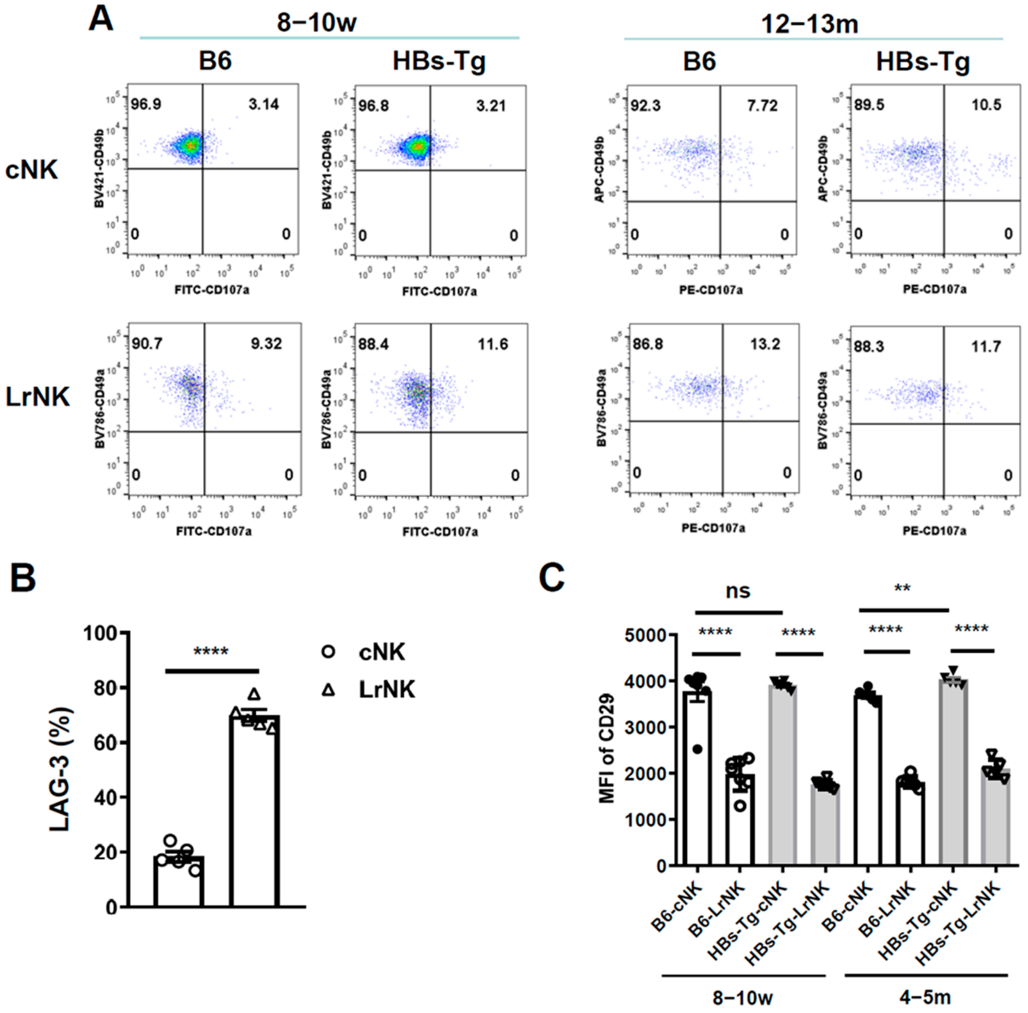

Figure S4. Expression levels of surface CD107a, Lag-3 and CD29 on intrahepatic cNK cells compared with LrNK cells in HBs-Tg mice and WT B6 mice. MNCs were isolated from livers of 8-10w-old, 4-5m-old and 12-13m-old HBs-Tg mice and control WT B6 mice, and then analyzed by flow cytometry. cNK cells ($CD3^-NK1.1^+CD49b^+CD49a^-$) and LrNK cells ($CD3^-NK1.1^+CD49b^-CD49a^+$) were gated to analyze the expressions of surface receptors for galectin-3. (**A**) Expression of surface CD107a. Percentages of positive population were shown. (**B**) Expression of Lag-3. HBs-Tg mice (6-7m-old) were observed. Percentages of positive population were shown. (**C**) Expression of CD29. MFI was shown. Data are shown as mean ± SEM. Student's *t* test was used. No significant statistical difference was defined as ns. ** P<0.01, **** P<0.0001 compared to the control group.

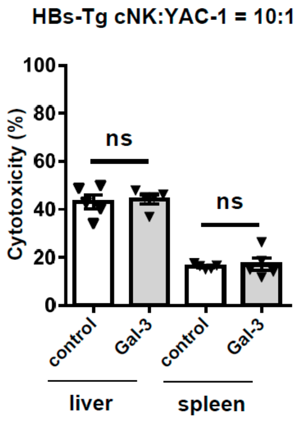

Figure S5. Galectin-3 did not affect the cytotoxicity of hepatic and splenic cNK cells against YAC-1 tumor cells. MNCs were isolated from livers and spleens of 4-5m-old HBs-Tg mice, and then cNK

cells were purified by MACS, respectively. cNK cells (1×10^5) were plated and cultured with Gal-3 protein (1 μg/mL). YAC-1 cells (1×10^4) stained with Far Red were used as the target cells. Data are shown as mean ± SEM. Student's *t* test was used. No significant statistical difference was defined as ns.

Reference

47. Jaruga B, Hong F, Kim WH, Gao B. IFN-gamma/STAT1 acts as a proinflammatory signal in T cell-mediated hepatitis via induction of multiple chemokines and adhesion molecules: a critical role of IRF-1. Am J Physiol Gastrointest Liver Physiol 2004;287:G1044-1052.
