# Supplementary figures and images for: Galectin-3-ITGB1 Signaling Mediates Interleukin 10 Production of Hepatic Conventional Natural Killer Cells in Hepatitis B Virus Transgenic Mice and Correlates with Hepatocellular Carcinoma Progression in Patients
Source: Viruses. 2024 May 7;16(5):737. doi: 10.3390/v16050737 (PMC11125742; doi:10.3390/v16050737)

Liver

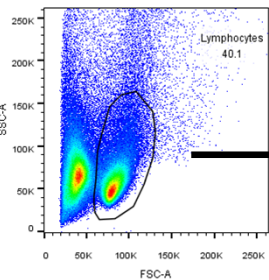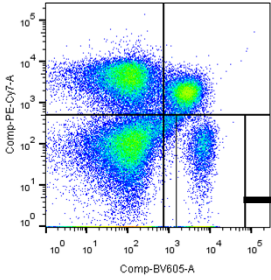

B6

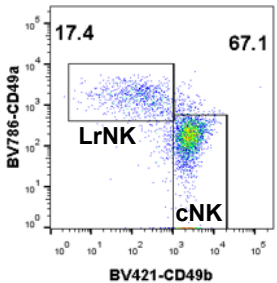

HBs-Tg

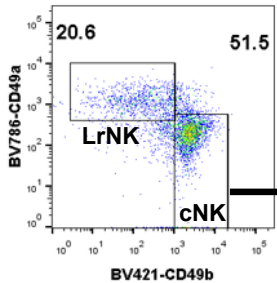

Phenotypes  
& functions

Spleen

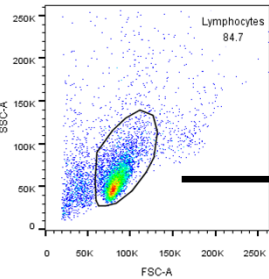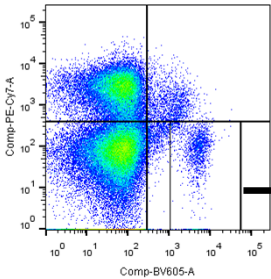

B6

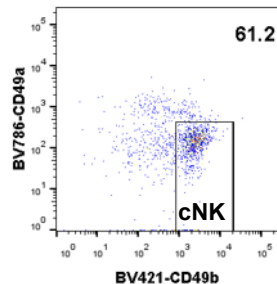

HBs-Tg

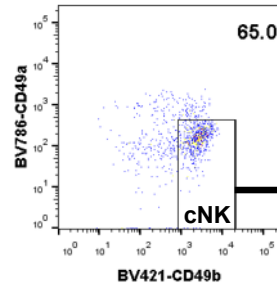

Phenotypes  
& functions

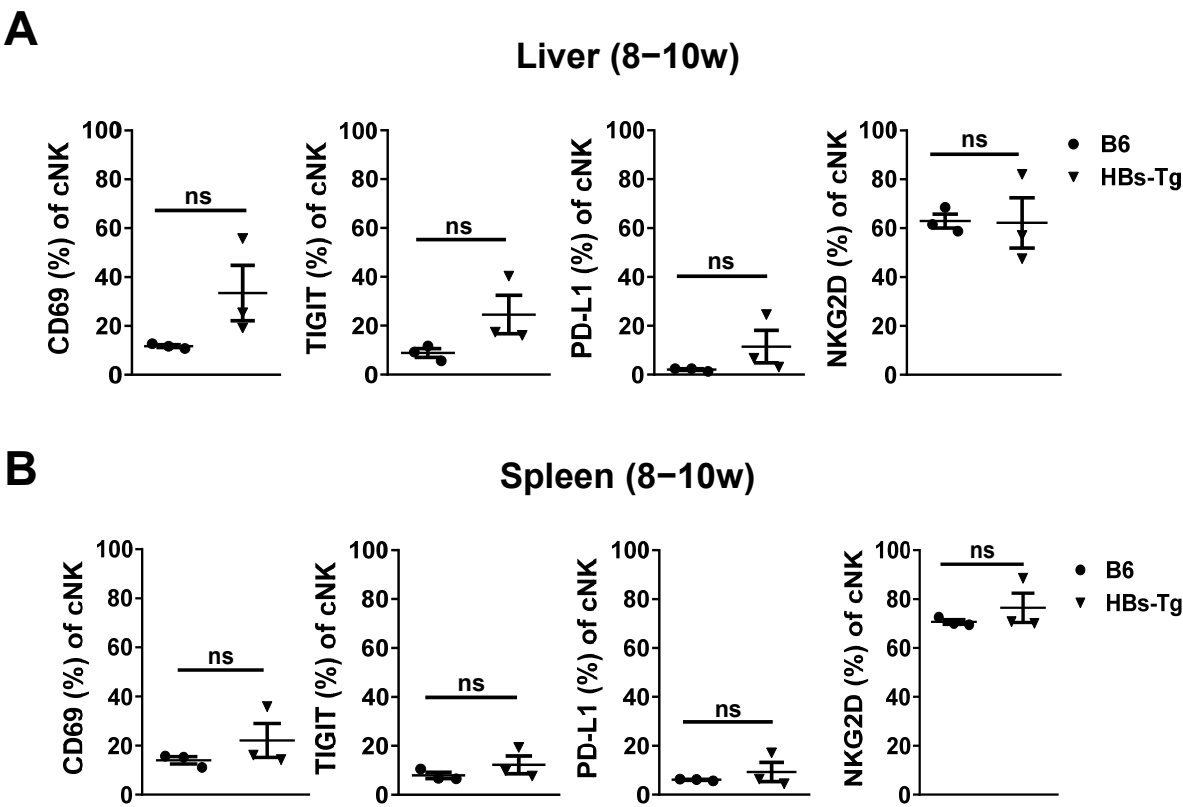

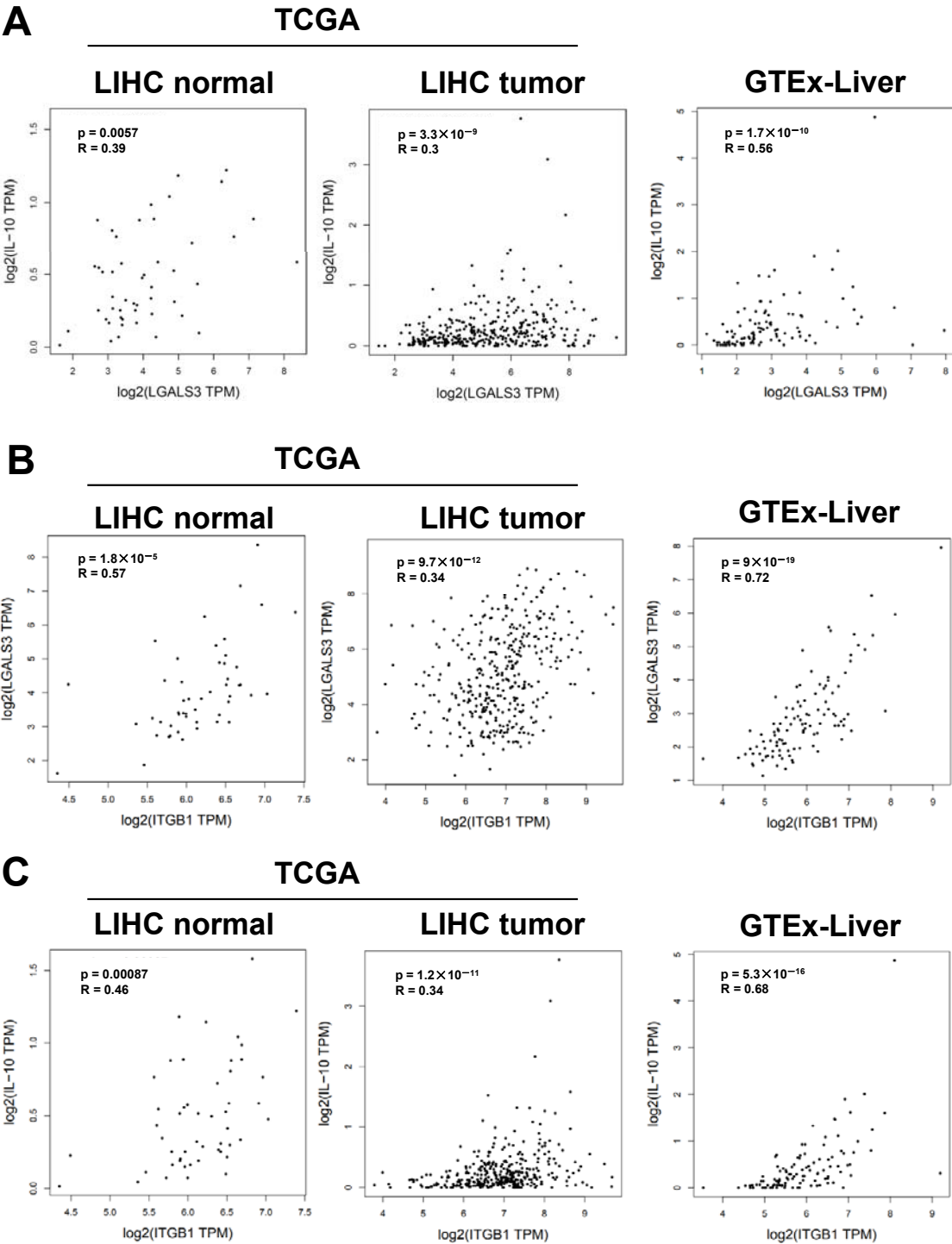

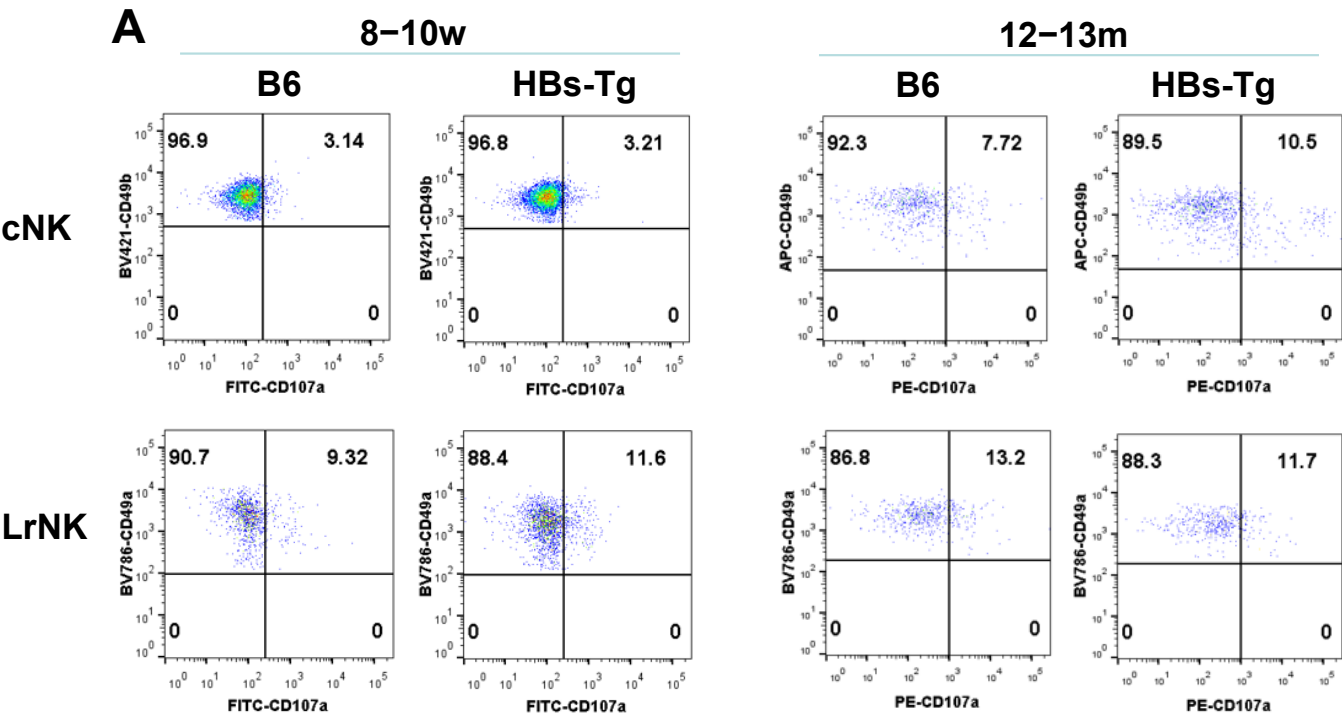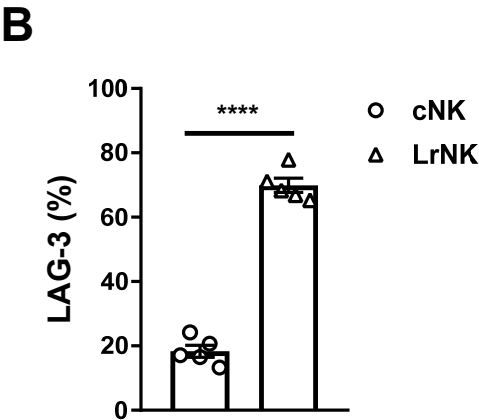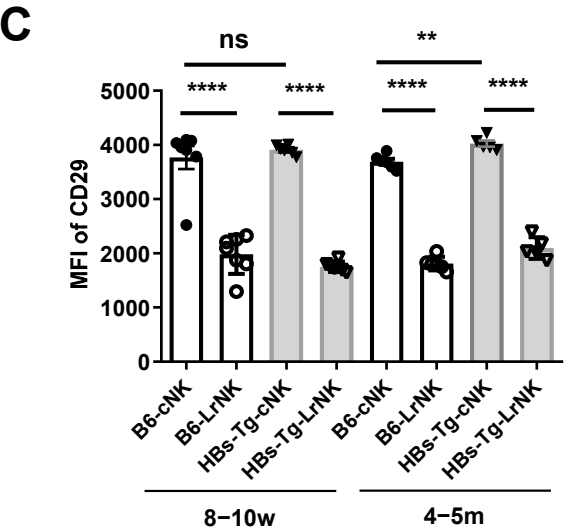

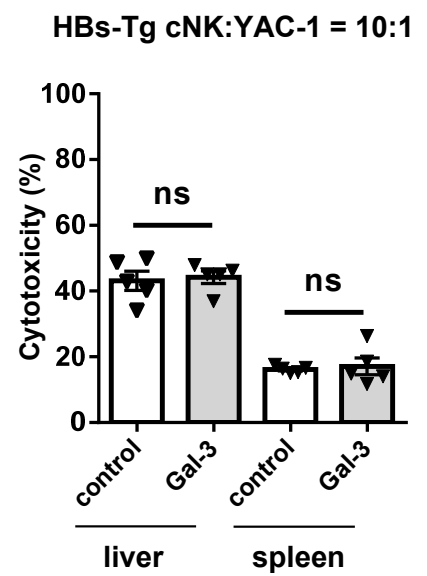

Supplement: Supplementary file 1 [file viruses-16-00737-s001.zip › Supplemental Figure-Chen YY-20240506-2.pdf]
